# The emerging economic evidence and methods used to evaluate clinical registries: a systematic scoping review protocol

**DOI:** 10.1136/bmjopen-2025-100644

**Published:** 2025-06-24

**Authors:** Kalpa Pisavadia, Ned Hartfiel, Limssy Varghese, Anne Krayer, Gemma Hobson, Rebecca Masters, Rob Poole, Catherine Robinson, Rhiannon Tudor Edwards, Emily Bebbington

**Affiliations:** 1 Centre for Health Economics and Medicines Evaluation, Bangor University, Bangor, UK; 2 Centre for Mental Health and Society, Bangor University, Bangor, UK; 3 Public Health Wales, NHS Wales, Cardiff, UK; 4 Social Care and Society, The University of Manchester Faculty of Biology Medicine and Health, Manchester, UK

**Keywords:** REGISTRIES, Health, HEALTH ECONOMICS, PUBLIC HEALTH

## Abstract

**Abstract:**

**Introduction:**

A clinical registry is a systematically collected database of health-specific information about a patient population. Clinical registries can be used for a variety of purposes including surveillance, monitoring of outcomes and patient care. The establishment and maintenance of clinical registries come with a significant cost. This scoping review aims to identify the methods used to economically evaluate clinical registries including their costs and benefits.

**Methods:**

This systematic scoping review protocol has been developed in accordance with the Preferred Reporting Items for Systematic Review and Meta-Analysis Protocols (PRISMA-P) guidelines. The final review will be reported following the Preferred Reporting Items for Systematic Reviews and Meta-Analyses extension for Scoping Reviews (PRISMA-ScR) checklist. The electronic databases Medline, Embase, Cochrane Library and The Cumulative Index to Allied Health Literature(CINAHL) database will be searched. Relevant national organisation websites will be searched to identify empirical studies within grey literature. The inclusion criteria include studies that economically evaluate clinical registries and are published in the English language from inception to February 2025. Two reviewers will independently screen 100% of titles and abstracts and full texts of studies for inclusion. Data will be extracted from eligible studies prior to being assessed for quality using a multi-tool approach.

**Ethics and dissemination:**

The findings of this review will be published in an international peer-reviewed journal. They are likely to be of interest to custodians of existing clinical registries and to those wishing to establish or evaluate clinical registries.

**Keywords**

Clinical registries, economic evaluation, costs, cost-effectiveness, health economics, registry based studies

**STRENGTHS AND LIMITATIONS OF THIS STUDY**

- This study will synthesise evidence on methods for the economic evaluation of clinical registries, an area that remains underexplored in registry research despite the recognised high costs of running a clinical registry.
- The protocol was developed in accordance with Preferred Reporting Items for Systematic Review and Meta-Analysis Protocols (PRISMA-P) guidelines. The completed review will be reported following the Preferred Reporting Items for Systematic Reviews and Meta-Analyses extension for Scoping Reviews (PRISMA-ScR) checklist.
- A tailored and comprehensive assessment of reporting quality, methodological robustness and potential bias will be conducted using a multi-tool approach.
- Relevant organisational websites will be searched to identify empirical studies within grey literature to increase the likelihood of identifying all relevant literature.
- Results will be restricted to English language studies, which may exclude some relevant articles.

## Introduction

 Clinical registries are systematically collected databases of health-specific information.^1^ Registries are implemented globally to measure the health and well-being of populations. The WHO defined a clinical registry as a disease-specific permanent record in 1967.^1 2^ Clinical registers have since been developed for surveillance purposes, tracking of patient outcomes, disease progression and treatment effectiveness. They have also been used to assess the quality of care, identify areas for improvement, inform research and develop evidence-based guidelines.^3^ Data can be collected from a range of settings including primary care, secondary care and community settings.

Clinical registries are sometimes referred to as disease registries, outcome registries or medical registries.^4^,^7^ They are utilised internationally in the fields of cancer care, congenital abnormalities, burn injuries, trauma, joint replacement and self-harm.^8^,^11^ There are estimated to be over 100 clinical registries in the UK.^12 13^ Registry systems are more common in high-income countries, which is attributed to the regulatory, ethical, technological and cost of maintaining these data collection systems.^14^

Registry data are commonly used to economically evaluate interventions, but economic evaluations of the clinical registry itself are less common despite the perceived high cost being a barrier to implementation and maintenance.^15^ Economic evaluations of clinical registries involve measuring both costs and health outcomes. Setting up and maintaining a clinical registry includes set-up costs (eg, information technology support), ongoing costs (eg, staff salaries) and development costs (eg, upgrading hardware and software).^16^ Registries are considered an alternative option to evaluate patient outcomes when clinical trials are impractical, as is often the case in social interventions.^17^ Though causality between registries and outcomes is difficult to establish, the existence of registries is associated with improvements in health outcomes.^18^ For example, the WHO recommends the establishment of a self-harm registry as a key government intervention for suicide prevention.^19^

Understanding how the costs and outcomes of clinical registries are evaluated can help inform clinical practice and funding decisions. A systematic review of economic evaluations focusing specifically on clinical quality registries, conducted in 2019, indicated that clinical quality registries can be cost-effective and can lead to significant returns on investment.^20^ However, the difficulty surrounding appropriate comparators resulted in uncertainty on the extent of patient improvement and cost savings of registries.^20^ As the 2019 review only included clinical quality registries, further exploration is necessary to identify the economic evidence and the methods used to evaluate all types of clinical registries. For the purpose of this review, we define a clinical registry as any systematically collected database of health-specific information that is referred to as a clinical registry by the authors of the included study.

## Study objectives

1. Determine the types of clinical registries that have undergone economic evaluation.
2. Identify the types of economic evaluations conducted on clinical registries.
3. Ascertain the quality of methods used to quantify the costs and benefits of clinical registries.
4. Summarise the available economic evidence of clinical registries.
5. Identify the implications for future research.

## Methods

This systematic scoping review protocol has been developed in accordance with the methodological frameworks from Arksey and O’Malley and the Joanna Briggs Institute (JBI), which provide widely accepted guidance for conducting scoping reviews.^21 22^ This protocol is reported in line with the Preferred Reporting Items for Systematic Review and Meta-Analysis Protocols checklist (PRISMA-P).^23^ The completed review will be reported using the Preferred Reporting Items for Systematic Reviews and Meta-Analyses extension for Scoping Reviews (PRISMA-ScR) checklist.^24^

### Eligibility criteria

The eligibility criteria for included articles are defined according to the Population, Intervention, Comparator, Outcomes (PICO) framework (table 1).

**Table 1:** Eligibility criteria (Population, Intervention, Comparator, Outcomes, Study design)

	Inclusion criteria	Exclusion criteria
Population and setting	All populations and settings are eligible for inclusion	Not applicable
Intervention/exposure	All clinical registries, defined as any systematically collected database of health-related information explicitly referred to as a ‘clinical registry’ in the study	Other database systems, for example, electronic health record systems
Comparison	Comparison groups may include different types of registries or historical patient data collected prior to the creation of the clinical registry. Studies without a comparison group will still be eligible for inclusion	Not applicable
Outcomes	Economic outcomes reported in evaluations of clinical registriesOutcomes may include healthcare resource use, the costs of creating and maintaining a clinical registry and other related costs	All studies that do not include any type of economic evaluation
Study designs	Any economic evaluation of a clinical registry.This may include any method of economic evaluation including cost-effectiveness analyses, cost-utility analyses, cost-benefit analyses, cost-minimisation analyses and return on investment analysisStudies of clinical registries reporting on costsSystematic reviews and reports of economic evaluations of clinical registries	All studies that do not include any type of economic evaluation or cost analysis
Countries	All countries included	There are no geographic or cultural restrictions
Language of publication	English language studies only	Full-text publications not available in the English language
Publication date	Inception to February 2025	No restrictions
Publication type	Published primary research studies, systematic reviews, grey literature (eg, government reports)	Theses, published abstracts

### Search strategy

A preliminary search of Google Scholar and PubMed was conducted using a combination of keywords and Medical Subject Headings (MeSH) terms such as registries, cost-benefit analysis and cost-effectiveness analysis. This preliminary search aimed to explore the breadth of clinical registries and economic evaluation terms. The search strategy was then designed in the Medline database to include key terms from these initial findings. The results were reviewed to ensure that previously identified articles of interest were captured. See online supplemental appendix 1 for the full Medline search strategy, which will be adapted for other bibliographic databases used in this review.

### Study records

The period searched will be from inception until February 2025, when the searches will be conducted. Searches will be conducted using the following databases: Medline (Ovid), Embase (Ovid), CINAHL (EBSCO) and Cochrane Library. Relevant national organisation websites will be searched to identify grey literature (eg, Australian Commission on Safety and Quality in Health Care, National Quality Registry Network and National Health Service (NHS) list of national clinical databases, registries and audits), which will be included if they are empirical studies that address the central research question of this scoping review.

### Study selection process

Two reviewers (KP and NH) will independently complete title and abstract screening and full-text article screening. Any disagreements between the two reviewers will be settled through consensus or consultation with a third reviewer (EB). The reason for full-text article exclusion will be recorded in Covidence based on the following hierarchy:

- Duplicate.
- Not in English.
- Non-human study.
- Not a clinical registry.
- Not an economic evaluation of a clinical registry.
- Not a cost analysis of a clinical registry.

A PRISMA flow diagram will be used to document the study selection process.^25^

### Data items

Data extraction items will include the following: citation, country, study aim, name of registry, type of registry, condition of interest, coverage of the registry (eg, national, regional and single-hospital) and setting (eg, hospital, primary care and school). Study design, type of economic evaluation, sample size, comparator/control, time period of study, length of follow-up, stakeholders of benefit, overall findings of the economic evaluation and knowledge gaps will also be captured.

Data items relevant to economic evaluations and cost studies will include the analytical approach (eg, trial-based or model-based), perspective of analysis, types of costs measured (including differentiation of direct costs, such as staffing and infrastructure, and indirect or in-kind costs, such as clinician time, where reported), currency and cost year, discounting, sensitivity analysis and uncertainty and confounding factors.

Outcomes will include primary outcomes, with differentiation between benefits related to quality improvement activities and secondary benefits, such as use in research, policy development or registry-based trials.

Where possible, data on the broader contributions of clinical registries, including their use in research, epidemiological analysis and service planning, will be extracted. Where such contributions are not formally quantified in economic evaluations, this limitation will be noted in the synthesis.

Data extraction will be completed in Covidence using an online data entry form. The extracted data will be downloaded for further analysis.

### Assessment of methodological quality

Quality appraisal of included studies will be conducted using a multi-tool approach based on study type. The Consolidated Health Economic Evaluation Reporting Standards 2022 statement (CHEERS) checklist will be used to assess the reporting quality of all economic evaluations.^26^ For studies focusing on costs only, the consensus-based checklist for the critical appraisal of cost-of-illness studies will be used to evaluate methodological rigour specific to this study type.^27^

To assess the methodological quality and risk of bias of full economic evaluations, we will use the Joanna Briggs Institute (JBI) Critical Appraisal Checklist for Economic Evaluations.^28^ The JBI checklist will also be applied to cost analysis studies and other partial economic evaluations. Items that are not applicable (eg, those requiring outcome or effectiveness data) will be clearly noted and justified, recognising the limitations of applying a full evaluation tool to partial studies. In order to address the limitations of this tool within cost analysis studies, methodological considerations from the consensus-based checklist for the critical appraisal of cost-of-illness studies will be used to supplement the appraisal of cost analysis studies. This multi-tool approach allows for a tailored and comprehensive assessment of reporting quality, methodological strength and potential bias across different types of economic studies.

### Synthesis

Results will be presented narratively and organised thematically, supplemented with descriptive tables. A meta-analysis is unlikely to be conducted due to the expected heterogeneity across the included studies. Results will be synthesised according to the study objectives and presented in accordance with the PRISMA-ScR checklist.^24^

### Patient and public involvement

Patients or the public were not involved in the design or reporting of this research protocol.

## Ethics and dissemination

Ethical approval is not required for this review. Results will also be disseminated more widely through publication in a peer-reviewed journal. They are likely to be of interest to custodians of existing clinical registries, as well as those who wish to establish a new clinical registry.

## Supplementary material

10.1136/bmjopen-2025-100644 online supplemental file 1
