# Supplementary material for: The emerging economic evidence and methods used to evaluate clinical registries: a systematic scoping review protocol
Source: BMJ Open. 2025 Jun 24;15(6):e100644. doi: 10.1136/bmjopen-2025-100644 (PMC12198832; doi:10.1136/bmjopen-2025-100644)
Supplement: online supplemental file 1 [file bmjopen-15-6-s001.docx]

**APPENDIX 1:** **Search strategy**

Ovid MEDLINE(R) and In-Process, In-Data-Review & Other Non-Indexed Citations <1946 to February 06, 2025>

| 1 | exp registries/ | 126502 |
| --- | --- | --- |
| 2 | Registries.mp. | 140371 |
| 3 | (("Registry" or "Registries" or "register" or "registers") adj5 ("cost" or "cost analysis")).mp. | 525 |
| 4 | 1 or 2 or 3 | 149875 |
| 5 | exp Cost-Benefit Analysis/ | 96757 |
| 6 | Cost-benefit.ti,ab,kf. | 14087 |
| 8 | Cost-eff*.ti,ab,kf. | 210616 |
| 9 | Cost-utility.ti,ab,kf. | 6930 |
| 10 | Cost-consequence.ti,ab,kf. | 460 |
| 11 | return on investment.ti,ab,kf. | 2760 |
| 12 | Budget impact.ti,ab,kf. | 2346 |
| 13 | 6 or 8 or 9 or 10 or 11 or 12 | 265077 |
| 14 | 4 and 13 | 2307 |
| 15 | limit 14 to english language | **2227** |
