# Supplementary material for: The emerging economic evidence and methods used to evaluate clinical registries: a systematic scoping review protocol
Source: BMJ Open. 2025 Jun 24;15(6):e100644. doi: 10.1136/bmjopen-2025-100644 (PMC12198832; doi:10.1136/bmjopen-2025-100644)
Supplement: Supplementary file 2 [file bmjopen-2025-100644.reviewer_comments.pdf]

PEER REVIEW HISTORY

BMJ Open publishes all reviews undertaken for accepted manuscripts. Reviewers are asked to complete a checklist review form and are provided with free text boxes to elaborate on their assessment. These free text comments are reproduced below.

ARTICLE DETAILS

Title (Provisional)

The emerging economic evidence and methods used to evaluate clinical registries: a systematic scoping review protocol.

Authors

Pisavadia, Kalpa; Hartfiel, N; Varghese, Limssy; Krayer, Anne; Hobson, Gemma; Masters, Rebecca; Poole, Rob; Robinson, Catherine; Edwards, Rhiannon Tudor; Bebbington, Emily

---

VERSION 1 - REVIEW

**Reviewer**	**1**
**Name**	**Ahern, Susannah**
**Affiliation**	**Monash University Department of Epidemiology and Preventive Medicine**
**Date**	**13-Apr-2025**
**COI**	**None**

Thank you for this protocol for a systematic scoping review of the economic evaluation of clinical registries. The rationale and justification for the study is sound, and based on the ongoing cost of clinical registries. Economic evaluation requires identifying costs vs benefits including health outcomes. The most recently conducted systematic review in 2019 noted difficulties in estimating costs and health outcomes. This review aims to include all clinical registries, not just clinical quality registries.

I would recommend that the data extracted should also allow for differentiation of direct costs (e.g. costs associated with registry staff and infrastructure) as well as indirect or in-kind costs such as clinician time in entering data into the registry database, if these are listed. I would also recommend that the data extracted relating to benefits and outcomes also note whether they are benefits related to the primary quality improvement activity, as well as secondary benefits such as research or e.g. registry based trials. Registries are increasingly undertaking analysis to support a range of research, epidemiological and planning activities, that ideally would be captured in an economic analysis if possible. If this is not possible nor appropriate the protocol should state this.

I would recommend that the protocol defines what a clinical registry is for the purpose of this systematic review e.g. should the 'registry' associated with the review have pre specified inclusion criteria or is any study that refers to a 'clinical registry' suitable for inclusion. This is referred to briefly in table 1, but it is likely that a clearer definition will be needed. I note that NHS has a List of national clinical databases, registries and audits. Will this list be used to include or exclude specific registries and therefore studies?

The comparison groups may include alternative data sources to registries, such as administrative patient data.

**Reviewer**	**2**
**Name**	**Gogovor, Amédé**
**Affiliation**	**Université Laval**
**Date**	**27-Apr-2025**
**COI**	**None**

This manuscript presents aimed at identifying the methods and economic evidence used to evaluate clinical registries. The study will an important contribution, given the substantial cost and under-evaluated economic impact of clinical registries in healthcare systems worldwide.

Please find below my comments to help improve the manuscript.

-My main comment is related to the use of the different to guide the written of the protocol, the conduct of the scoping review, and the reporting of the scoping review.

First, the authors should mention the methodological guideline to conduct the scoping review, e.g. Arksey, JBI

Second, the authors can refer to the Preferred Reporting Items for Systematic Reviews and Meta-Analyses extension for Scoping Reviews (PRISMA-ScR), as they did, or of the PRISMA-P to write the manuscript; and the PRISMA-ScR to report the methods and results of the scoping review

Third, the authors mentioned that the Consolidated Health Economic Evaluation Reporting Standards (CHEERS) checklist will be used to assess quality, but CHEERS is a reporting guideline, not a quality appraisal tool. I invite the authors to explore the website of the International Society for Pharmacoeconomics and Outcomes Research for methodological quality assessment of economic studies.

-Introduction: Page3, line 17, Important to insert e.g. here so I suggest “They are utilised internationally in the different field, e.g., cancer care, congenital abnormalities …

Methods/Data items: Page 6, lines 7-8, please clarify the double use of ‘setting’

Methods/Assessment of methodological quality: please see comments above

References: 22 & 24 are the same

VERSION 1 - AUTHOR RESPONSE

#	**Reviewers 1 comments**<br><br>Dr Susannah Ahern, Monash University Department of Epidemiology and Preventive Medicine	**Comment to reviewers**	**Location of amendment**
**1**	Thank you for this protocol for a systematic scoping review of the economic evaluation of clinical registries. The rationale and justification for the study is sound, and based on the ongoing cost of clinical registries. Economic evaluation requires identifying costs vs benefits including health outcomes. The most recently conducted systematic review in 2019 noted difficulties in estimating costs and health outcomes. This review aims to include all clinical registries, not just clinical quality registries.	Thank you for your thoughtful and encouraging feedback on our manuscript. We appreciate your recognition of the rationale and justification for this systematic scoping review. We agree that economic evaluation of clinical registries is a complex but important area of study, particularly in light of the challenges previously identified in estimating costs and health outcomes. We hope that by broadening the scope to include all clinical registries, our review will provide a more comprehensive understanding of the economic considerations involved and support future decision-making in this area.	No changes
**2**	I would recommend that the data extracted should also allow for differentiation of direct costs (e.g. costs associated with registry staff and infrastructure) as well as indirect or in-kind costs such as clinician time in entering data into the registry database, if these are listed.	Thank you for this helpful suggestion. We agree that distinguishing between direct and indirect (or in-kind) costs is important for a comprehensive economic evaluation. We confirm that our data extraction approach has included the differentiation of these cost types, where such details are reported in the included studies. We have now clarified this point in the manuscript to ensure this is clearly conveyed in the protocol.	Data items p. 5
**3**	I would also recommend that the data extracted relating to benefits and outcomes also note whether they are benefits related to the primary quality improvement activity, as well as secondary benefits such as research or e.g. registry based trials.	Thank you for this valuable suggestion. We agree that distinguishing between primary benefits related to the registry's core quality improvement activities and secondary benefits such as contributions to research or registry-based trials will enhance the depth and usefulness of our findings. We have updated the protocol to include this differentiation in the data	Data items p. 5

		extraction process and have clarified this in the Data items section.	
**4**	Registries are increasingly undertaking analysis to support a range of research, epidemiological and planning activities, that ideally would be captured in an economic analysis if possible. If this is not possible nor appropriate the protocol should state this.	Thank you for this insightful comment. We agree that registries increasingly contribute to a range of activities beyond their primary functions, and that these contributions should ideally be captured in economic evaluations. We have amended the protocol to reflect our intention to extract data on broader contributions of registries such as research, epidemiological analysis, and healthcare planning, where they are reported. We also acknowledge that these benefits may not always be quantified or included in the economic analyses and have noted this limitation in the protocol accordingly.	Data items p. 6
**5**	I would recommend that the protocol defines what a clinical registry is for the purpose of this systematic review e.g. should the 'registry' associated with the review have pre specified inclusion criteria or is any study that refers to a 'clinical registry' suitable for inclusion. This is referred to briefly in table 1, but it is likely that a clearer definition will be needed. I note that NHS has a List of national clinical databases, registries and audits. Will this list be used to include or exclude specific registries and therefore studies?	Thank you for your helpful comment. We agree that greater clarity around the definition of a clinical registry is important. For the purposes of this review, we have included any study that explicitly refers to a clinical registry. We did not use the NHS List of national clinical databases, registries, and audits to include or exclude specific registries or studies. However, we do plan on searching this for grey literature. We have now clarified this definition and our inclusion approach in the manuscript.	End of introduction, p. 3 and Table 1 under Intervention / exposure inclusion criteria
	**Reviewer 2 comments**<br><br>Dr Amédé Gogovor, Université Laval, VITAM Centre de Recherche en Sante Durable	**Comments to reviewers**	**Location of amendment**
**1**	This manuscript presents aimed at identifying the methods and economic evidence used to evaluate clinical registries. The study will an important contribution, given the substantial cost and under-evaluated economic impact of clinical registries in healthcare systems worldwide.	Thank you for your kind and supportive feedback. We are pleased that you recognise the potential contribution of our study. As you noted, clinical registries represent a significant investment within healthcare systems, and their economic impact remains under-evaluated. We hope that our review will help address this gap by providing clarity on the methods and evidence currently used in economic evaluations of clinical registries, thereby	No changes

		informing future research and policy development.	
**2**	First, the authors should mention the methodological guideline to conduct the scoping review, e.g. Arksey, JBI	Thank you for this helpful observation. While the protocol was not originally developed using the Arksey and O'Malley framework or the Joanna Briggs Institute (JBI) methodology, we recognise these as foundational and widely accepted approaches for conducting scoping reviews. We have now added a reference to these frameworks in the Methods section to acknowledge their relevance and to provide greater context for readers.	Methods<br>p. 4
**3**	Second, the authors can refer to the Preferred Reporting Items for Systematic Reviews and Meta-Analyses extension for Scoping Reviews (PRISMA-ScR), as they did, or of the PRISMA-P to write the manuscript; and the PRISMA-ScR to report the methods and results of the scoping review	Thank you for this helpful clarification. We appreciate your suggestion regarding the appropriate use of PRISMA extensions. We confirm that the PRISMA-P guidelines were used to inform the development of this scoping review protocol. While PRISMA-P is primarily intended for systematic review protocols, we acknowledge its relevance to protocol development more broadly. We have now clarified in the manuscript that the final review will be reported using the PRISMA-ScR checklist, while the current protocol has been informed by PRISMA-P where applicable.	Methods<br>P. 4<br><br>Abstract<br>p. 1<br><br>Strengths and limitations<br>p. 2
**4**	Third, the authors mentioned that the Consolidated Health Economic Evaluation Reporting Standards (CHEERS) checklist will be used to assess quality, but CHEERS is a reporting guideline, not a quality appraisal tool. I invite the authors to explore the website of the International Society for Pharmacoeconomics and Outcomes Research for methodological quality assessment of economic studies.	Thank you for this valuable comment. We acknowledge that the CHEERS checklist is a reporting guideline rather than a tool for assessing methodological quality. Since submitting the protocol, we have refined our approach to ensure a more robust and appropriate appraisal process. We will now use the CHEERS 2022 statement to assess the reporting quality of all economic evaluations, and the consensus-based checklist for the critical appraisal of cost-of-illness (COI) studies for studies focusing on COI.<br><br>To assess the risk of bias and methodological rigour of economic evaluations, we will use the Joanna Briggs Institute (JBI) Critical Appraisal Checklist for Economic Evaluations. This tool will be applied to all full economic evaluations included in the	Assessment of methodological quality<br>p. 6<br><br>Abstract<br>p. 1<br><br>Strengths and limitations<br>p. 2

		review. For cost analysis studies, we will also use the JBI checklist, but items not applicable to partial evaluations (e.g. those requiring outcome or effectiveness data) will be noted accordingly. Where appropriate, this will be supplemented by relevant methodological considerations for cost-of-illness studies. These changes have been reflected in the revised protocol.	
**5**	Introduction: Page3, line 17, Important to insert e.g. here so I suggest "They are utilised internationally in the different field, e.g., cancer care, congenital abnormalities …	Thank you for your observation. We appreciate the suggestion in rephrasing this sentence. However, we believe the current phrasing clearly presents a list of representative fields where clinical registries are utilised. As such, we have chosen to retain the original wording.	No changes
**6**	Methods/Data items: Page 6, lines 7-8, please clarify the double use of 'setting'	Thank you for pointing this out. We have removed the duplication.	Data items p. 5
**7**	References: 22 & 24 are the same	Thank you for pointing this out. We have removed the duplicate and renumbered the references accordingly.	Synthesis p.6
